# Noninvasive Evaluation of EGFR Expression of Digestive Tumors Using ^99m^Tc-MAG_3_-Cet-F(ab′)_2_-Based SPECT/CT Imaging

**DOI:** 10.1155/2022/3748315

**Published:** 2022-06-24

**Authors:** Dai Shi, Yiqiu Zhang, Zhan Xu, Zhan Si, Yuan Cheng, Dengfeng Cheng, Guobing Liu

**Affiliations:** ^1^Department of Nuclear Medicine, Zhongshan Hospital, Fudan University, Shanghai 200032, China; ^2^Institute of Nuclear Medicine, Fudan University, Shanghai 200032, China; ^3^Shanghai Institute of Medical Imaging, Shanghai 200032, China; ^4^Cancer Prevention and Treatment Center, Zhongshan Hospital, Fudan University, Shanghai 200032, China

## Abstract

**Purpose:**

This study is aimed at investigating the feasibility of cetuximab (Cet) F(ab′)_2_ fragment- (Cet-F(ab′)_2_-) based single photon emission tomography/computed tomography (SPECT/CT) for assessing the epidermal growth factor receptor (EGFR) expression in digestive tumor mouse models.

**Methods:**

Cet-F(ab′)_2_ was synthesized using immunoglobulin G-degrading enzyme of *Streptococcus pyogenes* (IdeS) protease and purified with protein A beads. The product and its in vitro stability in normal saline and 1% bovine serum albumin were analyzed with sodium dodecyl sulfate–polyacrylamide gel electrophoresis. The EGFR expression in the human colon tumor cell line HT29 and the human stomach tumor cell line MGC803 were verified using western blotting and immunocytochemistry. Cet-F(ab′)_2_ was conjugated with 5(6)-carboxytetramethylrhodamine succinimidyl ester to demonstrate its binding ability to the MGC803 and HT29 cells. Cet-F(ab′)_2_ was conjugated with NHS-MAG_3_ for ^99m^Tc radiolabeling. The best imaging time was determined using a biodistribution assay at 1, 4, 16, and 24 h after injection of the ^99m^Tc-MAG_3_-Cet-F(ab′)_2_ tracer. Furthermore, ^99m^Tc-MAG_3_-Cet-F(ab′)_2_ SPECT/CT was performed on MGC803 and HT29 tumor-bearing nude mice.

**Results:**

HT29 cells had low EGFR expression while MGC803 cell exhibited the high EGFR expression. Cet-F(ab′)_2_ and intact cetuximab showed similar high binding ability to MGC803 cells but not to HT29 cells. Cet-F(ab′)_2_ and ^99m^Tc-MAG_3_-Cet-F(ab′)_2_ showed excellent in vitro stability. The biodistribution assay showed that the target to nontarget ratio was the highest at 16 h (17.29 ± 5.72, *n* = 4) after tracer injection. The ^99m^Tc-MAG_3_-Cet-F(ab′)_2_-based SPECT/CT imaging revealed rapid and sustained tracer uptake in MGC803 tumors rather than in HT29 tumors with high image contrast, which was consistent with the results in vitro.

**Conclusion:**

SPECT/CT imaging using ^99m^Tc-MAG_3_-Cet-F(ab′)_2_ enables the evaluation of the EGFR expression in murine EGFR-positive tumors, indicating the potential utility for noninvasive evaluation of the EGFR expression in tumors.

## 1. Introduction

Gastric cancer and colon cancer are common digestive system tumors, with incidence rates ranked fifth [1] and third [2] among that of all tumors, respectively. Gastric cancer and colon cancer are both among the top five causes of tumor-related death. Many patients are already at the advanced stages when diagnosed and are therefore usually unsuitable for radical surgery. Advances in the assessment of the target point in targeted therapy have contributed to increased treatment effectiveness and improved survival of patients with cancer over the past decades. Epidermal growth factor receptor (EGFR), the receptor for EGF cell proliferation and signal transduction, is related to the inhibition of tumor cell proliferation, angiogenesis, tumor invasion, metastasis, and apoptosis [3, 4]. Monoclonal antibody (mAb) treatment targeting EGFR has demonstrated high therapeutic efficacy in the clinic [5–7]. However, treatment response is always achieved only in patients with cancer with high EGFR expression.

Traditional computed tomography (CT) and magnetic resonance imaging (MRI) in tumor diagnosis and staging mainly reveal anatomic changes. The expression of specific molecules in a tumor is difficult to demonstrate. Single photon emission computed tomography/computed tomography (SPECT/CT) based on immunological probes (immuno-SPECT/CT) is a common noninvasive molecular imaging method that utilizes a radiolabeled antibody to visualize a specific marker [8–10]. The therapeutic effect of EGFR targeted treatment depends highly on the EGFR expression of the tumor. Although pathological results are the gold standard, the means of obtaining samples are typically invasive and inconvenient. Cetuximab (Cet), a US Food and Drug Administration- (FDA-) approved mAb, is widely used for treating digestive tumors with high EGFR expression. However, noninvasive methods that can efficiently classify patients with high-EGFR expression tumors for intensive EGFR targeted treatment are rare.

The intact antibody commonly has a molecular weight of about 150 kDa, which makes its metabolism in the blood very slow (its biological half-life (*T*_1/2_) is always >3 days) [11]. Therefore, it presents significant radiation problems for nuclear medicine immunoimaging. Enzymatic digestion can produce F(ab′)_2_ fragments (about 100 kDa) from an intact antibody to reduce the molecular weight but nevertheless retain the antigen-binding site and immunological binding activity of the intact antibody [9, 12]. In the present study, we fabricated ^99m^Tc-labeled cetuximab F(ab′)_2_ fragments (Cet-F(ab′)_2_) as a probe for biodistribution and SPECT/CT imaging assessment of the EGFR expression in murine models of digestive tumors.

## 2. Materials and Methods

### 2.1. Cell Culture

HT29 human colon cancer and MGC803 human stomach tumor cell lines were from Shanghai Zhong Qiao Xin Zhou Biotechnology Co., Ltd. (Shanghai, China) and incubated in Dulbecco's Modified Eagle's Medium (Servicebio, Wuhan, China) containing 10% heat-inactivated fetal bovine serum (FBS, Gibco, Waltham, MA, USA).

### 2.2. Mice and Reagents

All animal studies were conducted in accordance with protocols approved by the Animals Ethics Committee of Zhongshan Hospital, Fudan University. Eight-week-old male BALB/c nude mice (20–22 g) were from Charles River (Beijing, China). Anti-EGFR mAb (#ab52894) was from Abcam (Cambridge, UK). Horseradish peroxidase conjugated goat anti-rabbit IgG (H + L) (#GB23303), Cy3-conjugated goat anti-rabbit IgG (H + L) (#GB21303), and DAPI (#G1012) were from Servicebio (Wuhan, China). Anti-*β*-actin mAb (#4970) was from Cell Signaling Technologies (Boston, MA, USA). Cetuximab (#A2000) was from Selleck (Shanghai, China). Immunoglobulin G-degrading enzyme of Streptococcus pyogenes (IdeS) protease (#20412ES84) was from Yeasen (Shanghai, China).

### 2.3. Western Blotting

The MGC803 and HT29 cells were seeded in 6-well plates and grown to 70% confluence. The cells were lysed using radioimmunoprecipitation assay lysis buffer plus 1 mM PMSF (Servicebio, Wuhan, China) at 4°C for 30 min. The supernatant was collected, and the protein concentration was quantified by a spectrophotometer. Then, the proteins were denatured with protein loading buffer at 100°C for 10 min. Total protein (20 *μ*g) was loaded into gels (EpiZyme, Shanghai, China) with Muticolor Prestained Protein Ladder (EpiZyme, Shanghai, China). Electrophoresis was performed at 80 V for 30 min and then 120 V for 60 min. All proteins were then transferred to a PVDF membrane. The membrane was blocked with skim milk (5%) blocking buffer for 2 h at room temperature (20°C) and incubated overnight at 4°C with rabbit anti-EGFR antibodies (1 : 1000 dilution, Abcam) and rabbit anti-*β*-actin mAb (1 : 10,000 dilution, Cell Signaling Technology). Next, the membrane was washed three times with TBS-Tween 20 and incubated with goat anti-rabbit IgG antibodies (1 : 5000, Servicebio, Wuhan, China) for 2 h at room temperature. The washed membrane was scanned and quantitatively analyzed using a Tanon 4200 imaging system (Tanon, Shanghai, China). The EGFR expression was analyzed and normalized to *β*-actin protein for comparison between the MGC803 and HT29 cells using ImageJ 1.44p (National Institutes of Health, Bethesda, MA, USA).

### 2.4. Preparation of Cet-F(ab′)_2_

Cetuximab was incubated with IdeS protease for 30 min at 37°C in digestion buffer (50 mM sodium phosphate, 150 mM NaCl, pH 6.6). The digested products were incubated with protein A beads for 1 h and centrifuged. The Fc portion attached to the beads was removed in the sediment while the purified Cet-F(ab′)_2_ remained in the supernatant. The Cet-F(ab′)_2_ and cetuximab were evaluated by sodium dodecyl sulfate-polyacrylamide gel electrophoresis (SDS-PAGE) on a 4–15% gel under 150 V for 1 h. The stability of Cet-F(ab′)_2_ in PBS and 1%BSA was evaluated through SDS-PAGE.

### 2.5. Immunocytochemistry

Cet-F(ab′)_2_ and cetuximab were conjugated with 5(6)-carboxytetramethylrhodamine succinimidyl ester (5(6)-TAMRA, Xi'an Ruixi Biological Technology, Xi'an, China) for 2 h, with a 1 : 3 molar ratio of Cet-F(ab′)_2_ to 5(6)-TAMRA/cetuximab to 5(6)-TAMRA in carbonate buffer (pH 9.0). The fluorescent product was purified using PD-10 columns by removing excess dye. After MGC803 cells had been cultured in 6-well plates to 40% confluence, they were incubated with 66.67 nmol/L TAMRA-Cet-F(ab′)_2_ or TAMRA-cetuximab overnight. Images were captured using an Olympus imaging system (Olympus, Tokyo, Japan).

### 2.6. Preparation of ^99m^Tc-MAG_3_-Cet-F(ab′)_2_

**Figure 1:**
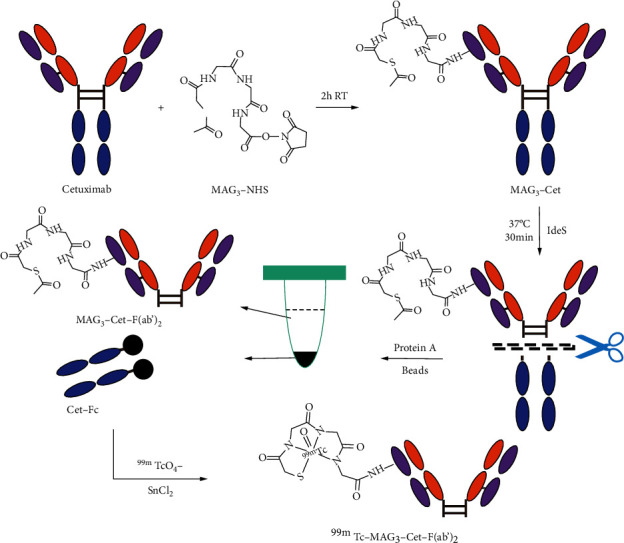
Schematic diagram illustrating the synthesis of ^99m^Tc-MAG_3_-Cet-F(ab′)_2_. Please note that this figure only illustrates the synthesis of the radiolabel, and not that only one MAG_3_ molecule attaches to one cetuximab moiety.

Figure 1 shows the synthetic route of ^99m^Tc-MAG_3_-Cet-F(ab′)_2_. In brief, cetuximab was incubated with MAG_3_ for 2 h at room temperature in carbonate buffer (pH 9.0). The molar ratio of cetuximab to MAG_3_ was 1 : 5, according to previous described methods [13–15]. The MAG_3_-Cet was purified using a Zeba Spin Desalting Column 7 K MWCO (Thermo Fisher Scientific, Waltham, MA, USA). MAG_3_-Cet-F(ab′)_2_ was prepared using IdeS protease and purified by removing the Fc portion using protein A beads (Epizyme Biomedical Technology, Shanghai, China). Figure S1 shows the characterization of MAG_3_-Cet and MAG_3_-Cet-F(ab′)_2_ by SDS-PAGE (Supplementary File). The cheator-to-antibody ratio of the product was determined by liquid chromatography-mass spectrometry (LC-MS) (Bioaccord, Waters, Milford, USA). In brief, MAG_3_-Cet was incubated with IdeS protease for 30 min at 37°C in digestion buffer (50 mM sodium phosphate, 150 mM NaCl, pH 6.6). The digested products were incubated with protein A beads for 1 h and centrifuged. The Fc portion attached to the beads was removed in the sediment while the purified MAG_3_-Cet-F(ab′)_2_ remained in the supernatant. Size-exclusion high performance liquid chromatography (SEC-HPLC) was performed to determine the radio purity of the product using an Agilent 1260 Infinity II Bio-Inert System (Agilent, Santa Clara, USA) with a Tosoh Bioscience TSK gel (G3000SWXL, 7.8 mm × 300 mm) at 20°C and a Bioscan B-FC-1000 radiation detector (energy range: 0–20 M cpm, Eckert & Ziegler Group, Hopkinion, USA). The mobile phase was PBS (pH 7.4). The flow rate was set at 0.5 mL/min. Sample was detected at 220 nm with a UV detector. For ^99m^Tc labeling, 100 *μ*g MAG_3_-Cet-F(ab′)_2_ was added to a combined solution of 45 *μ*L ammonium acetate (0.25 M) and 15 *μ*L tartrate buffer, and then no more than 25 *μ*L (approximately 5 mCi) ^99m^Tc-pertechnetate generator eluate was added. Immediately after vortexing, 3 *μ*L freshly prepared 1 mg/mL SnCl_2_∙2 H_2_O solution was added. The combined solution was incubated at room temperature for 1 h under vortexing. ^99m^Tc-MAG_3_-Cet-F(ab′)_2_ was purified from unlabeled reduced ^99m^Tc with PD-10 desalting columns. Radio-HPLC was performed to determine the radiochemical purity of ^99m^Tc-MAG_3_-cet-F(ab′)_2_ with the same machine, mobile phase, and flow rate as mentioned above.

### 2.7. Stability, Competition and Binding Assay of ^99m^Tc-MAG_3_-Cet-F(ab′)_2_

The stability of ^99m^Tc-MAG_3_-Cet-F(ab′)_2_ was determined in 1× phosphate-buffered saline (PBS) and 1% bovine serum albumin (BSA) for 1, 6, 12, and 24 h. The unfolding agent was a 1 : 2 (v/v) mixture of 0.9% saline and methanol. For the competition assay, 2 × 10^5^ MGC803 cells were seeded in 24-well plates with 1.25–1280 nM unlabeled cetuximab and 10 nM ^99m^Tc-MAG_3_-Cet-F(ab′)_2_ and incubated at 37°C for 60 min. The supernatant was discarded, and the cells were washed twice with iced 1× PBS and harvested for determination of radioactivity using a gamma counter. For the binding assay, 2 × 10^5^ MGC803 cells and HT29 cells each were seeded in 24-well plates. ^99m^Tc-MAG_3_-Cet-F(ab′)_2_ (0.1–4 nM) in PBS solution was added to the plates and incubated at room temperature for 1 h. Then, the supernatant was discarded while the cells were washed twice with iced 1× PBS and harvested for determination of radioactivity. The binding results including the maximum binding ability (*B*_max_) and the dissociation constant (*K*_*d*_) were obtained via GraphPad Prism (GraphPad Inc., La Jolla, CA, USA).

### 2.8. Mouse Model Preparation

All animal studies were performed in accordance with protocols approved by the Animals Ethics Committee of Zhongshan Hospital, Fudan University. Subcutaneous MGC803 and HT29 tumors were induced in 6-week-old male nude mice, by injecting their lower right flanks with 1 × 10^6^ tumor cells suspended in 200 *μ*L PBS. The tumors were monitored every other day.

### 2.9. Biodistribution and Micro-SPECT/CT Imaging

The MGC803 tumor-bearing mice (*n* = 16) were injected with 18.5 MBq (50 *μ*g) ^99m^Tc-MAG_3_-Cet-F(ab′)_2_. After 1, 6, 16, and 24 h, four mice from each group were sacrificed and dissected. Tumors, blood, and major tissues/organs (including heart, lung, liver, kidney, spleen, colon, stomach, bone, and muscle) were harvested and weighed. Sample tissue radioactivity was measured using a gamma counter. The radioactivity concentration of the tissue was expressed as the percentage injected dose per g (%ID/g), and the tumor to muscle (T/M) ratio was defined as the ratio of radioactivity that had accumulated in tumors to that in the contralateral muscle. The experiments were repeated three times.

Micro-SPECT/CT scanning was conducted using a Nano SPECT/CT scanner (BioScan, Washington DC, USA). ^99m^Tc-MAG_3_-Cet-F(ab′)_2_ (18.5 MBq/50 *μ*g/mouse) was injected into tumor-bearing mice via the tail vein. After 16 h, the mice were anesthetized via 2% isoflurane inhalation. CT was performed first with the following parameters: frame resolution, 256 × 512; tube voltage, 45 kVp; current, 0.15 mA; and exposure time, 500 ms/frame. Each scan spanned about 7 min. SPECT was performed after CT scanning with same bed position and the following parameters: four high-resolution conical collimators with 9-pinhole plates; energy peak, 140 keV; window width, 10%; resolution, 1 mm/pixel; matrix, 256 × 256; and scan time, 70 s/projection, 24 projections in all. Each mouse was scanned in 42 minutes on average. Three-dimensional ordered-subset expectation maximization images were reconstructed using the HiSPECT algorithm. Reconstructed SPECT/CT data were transferred to InVivoScope (Version 1.43, BioScan) for postprocessing.

### 2.10. Immunofluorescence

After deparaffinization and hydration, the HT29 and MGC803 tumor slices were incubated in 5% BSA in PBS buffer for 1 h. Then, the slices were incubated in rabbit anti-EGFR antibody (1 : 200) overnight at 4°C. After washing in PBS, the slices were stained with CY3-conjugated goat anti-rabbit antibody (1 : 100) for 1 h. Following three PBS washes, the nuclei were stained using DAPI. Images were captured using an Olympus imaging system.

### 2.11. Statistical Analysis

Data are presented as the means ± standard deviation (SD) derived from at least three independent experiments. The Student *t*-test was applied for intergroup comparisons using GraphPad Prism. All tests were 2-sided, and a statistical *P* value of <0.05 was considered statistically significant.

## 3. Results

### 3.1. MGC803 and HT29 Cell EGFR Expression Levels

**Figure 2:**
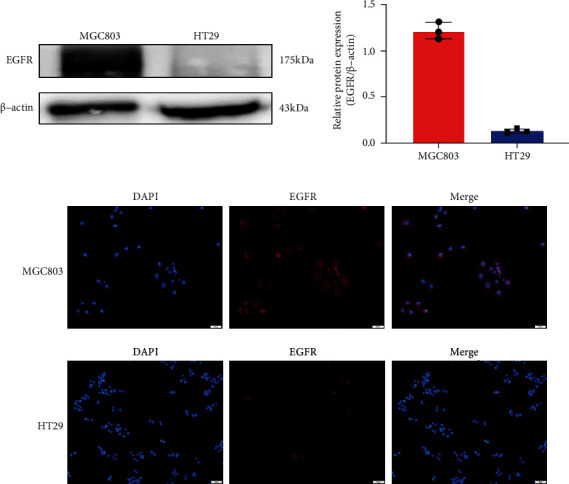
Evaluation of the EGFR expression in MGC803 and HT29 tumor cells. Western blots (a) and immunofluorescence of tumor cell (b) showed a higher EGFR expression level in MGC803 cells compared to HT29 cells.

The EGFR expression in MGC803 cells and HT29 cells was quantified by western blotting. MGC803 cells had a significantly higher relative expression ratio of EGFR/*β*-actin than HT29 cells in vitro (1.21 ± 0.10 vs. 0.13 ± 0.02, *P* < 0.01) (Figure 2(a)). Immunocytochemistry showed stronger fluorescence intensity in MGC803 cells, which was consistent with western blotting results (Figure 2(b)).

### 3.2. Molecular Weight, Binding Affinity, and Stability of Cet-F(ab′)_2_

**Figure 3:**
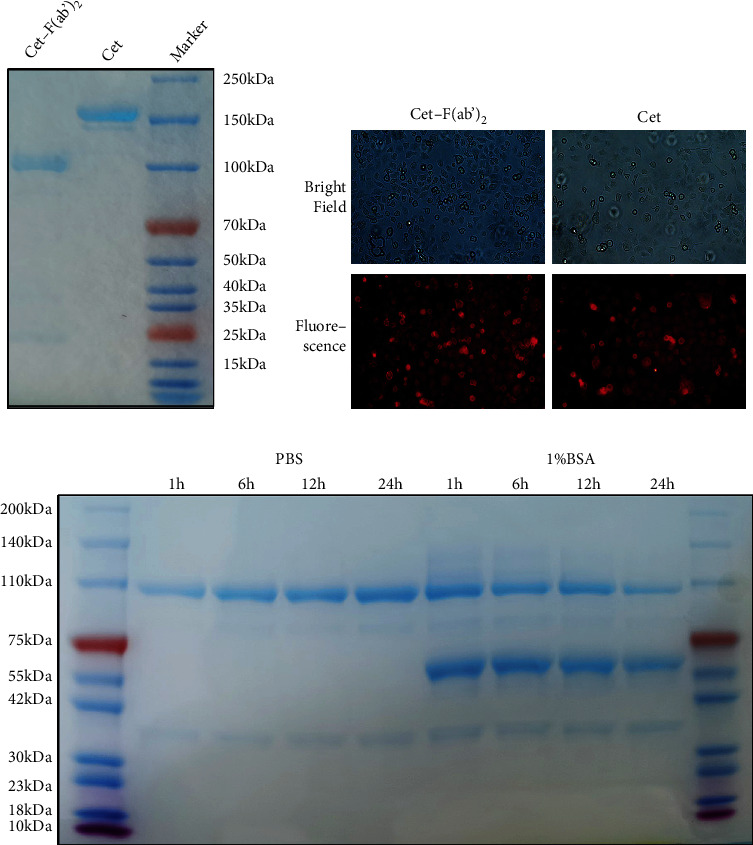
Verification of Cet-F(ab′)_2_ on its molecular weight, binding affinity to EGFR positive cells, and in vitro stability. (a) SDS-PAGE showing that the molecular weight of Cet-F(ab′)_2_ and cetuximab is 100 kDa and 150 kDa, respectively. (b) Immunocytochemistry showing high binding ability of Cet-F(ab′)_2_ and cetuximab to MGC803 cells. (c) SDS-PAGE illustrating the excellent stability of Cet-F(ab′)_2_ after incubation in PBS and 1% BSA over 24 h.

SDS-PAGE showed that the molecular weight of Cet-F(ab′)_2_ and cetuximab was about 100 kDa and 150 kDa, respectively (Figure 3(a)). Fluorescence microscopy showed that TAMRA-Cet-F(ab′)_2_ had similar binding affinity for MGC803 cells compared with TAMRA-Cet (Figure 3(b)). SDS-PAGE showed that Cet-F(ab′)_2_ had excellent stability following incubation in PBS or 1%BSA, with >90% remained intact until 24 h (Figure 3(c)).

### 3.3. Successful Preparation of MAG_3_-Cet-F(ab′)_2_ and ^99m^Tc-MAG_3_-Cet-F(ab′)_2_

**Figure 4:**
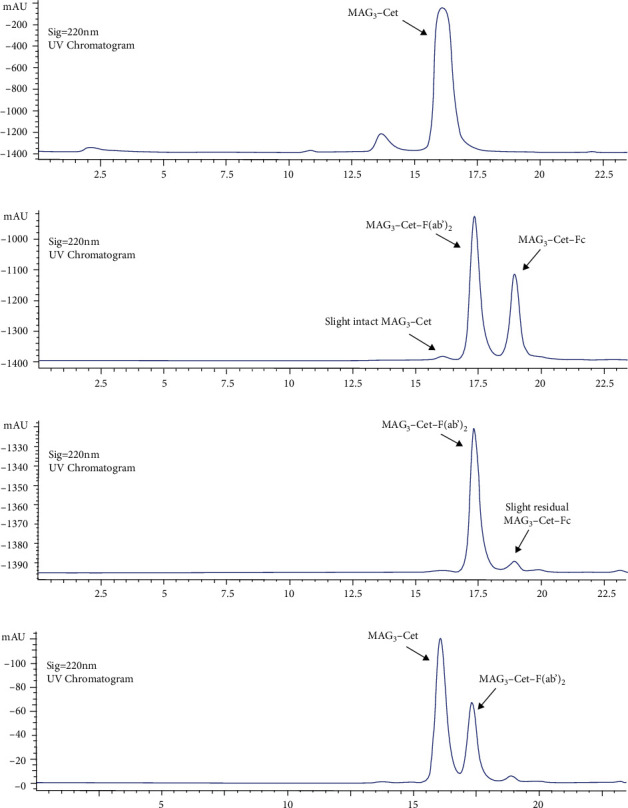
The HPLC results showing ultraviolet profiles of MAG_3_-Cet, MAG_3_-Cet-F(ab′)_2_ and MAG_3_-Cet-Fc. (a) The HPLC result of MAG_3_-Cet. (b) The HPLC result of MAG_3_-Cet-F(ab′)_2_ and MAG_3_-Cet-Fc after digesting MAG_3_-Cet with IdeS protease. (c) The HPLC result of the mixture from (b) but after reaction with protein A beads. The residual MAG_3_-Cet and most of the MAG_3_-Cet-Fc were removed by protein A beads. (d) The HPLC result of the mixture of (a) and (c).

**Figure 5:**
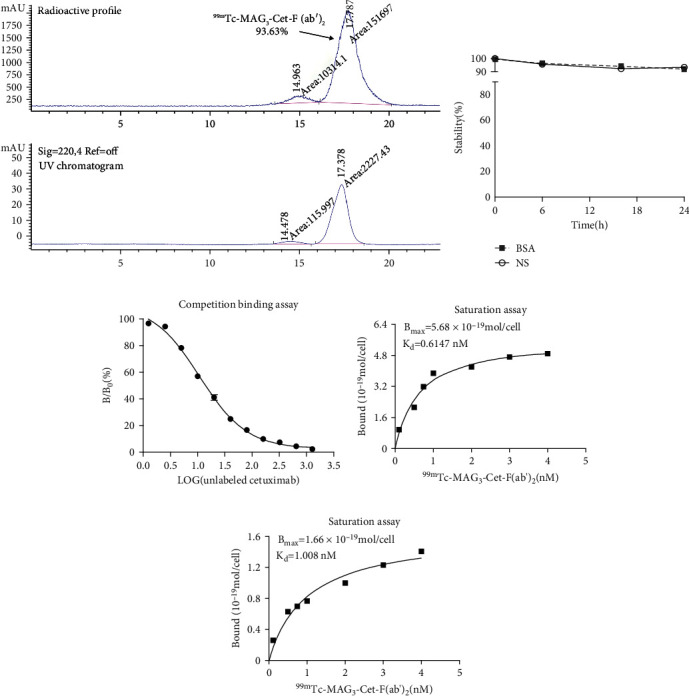
Characterization of ^99m^Tc-MAG3-Cet-F(ab′)_2_ in vitro. (a) The radio-HPLC result of ^99m^Tc-MAG_3_-Cet-F(ab′)_2_. (b) Stability assay of ^99m^Tc-MAG_3_-Cet-F(ab′)_2_ in 1% BSA and NS. (c) Competition binding assay between ^99m^Tc-MAG_3_-Cet-F(ab′)_2_ and unlabeled cetuximab to MGC803 cells (d, e). Representative saturation binding curve of ^99m^Tc-MAG_3_-Cet-F(ab′)_2_ binding to MGC803 cells (d) and HT29 cells (e), indicating its higher affinity to MGC803 cells (*B*_max_ = 5.68 × 10^−19^ mol ligands/cell, Kd = 0.6147 nM), compared to HT29 cells (*B*_max_ = 1.66 × 10^−19^ mol ligands/cell, Kd = 1.008 nM).

Characterization of successful MAG_3_-Cet-F(ab′)_2_ preparation using HPLC is shown in Figure 4. The overall conjugation ratio of MAG_3_ per Cet-F(ab′)_2_ identified by LC-MS was about 0.74 (Figure S2). Characterization of ^99m^Tc-MAG_3_-Cet-F(ab′)_2_ using SDS-PAGE can be found in Figure S3. As identified by SEC-HPLC, the radio purity ^99m^Tc-MAG_3_-Cet-F(ab′)_2_ before purification about 82.2%. After purification with PD-10 desalting column, the radiochemical purity was about 93.63% (Figure 5(a)). The specific activity and radioactive concentration of the final preparation were 1.48 MBq/*μ*g and 296 MBq/mL, respectively. The ^99m^Tc-MAG_3_-Cet-F(ab′)_2_ had excellent stability, with >90% remaining intact over 24 h in both normal saline (NS) and 1% BSA (Figure 5(b)). The competition binding assay showed that ^99m^Tc-MAG_3_-Cet-F(ab′)_2_ had excellent specificity for MGC803 tumor cells (Figure 5(c)). Unlabeled cetuximab with >1000-fold concentration almost blocked ^99m^Tc-MAG_3_-Cet-F(ab′)_2_ binding to MGC803 tumor cells (<3%). The fabricated ^99m^Tc-MAG_3_-Cet-F(ab′)_2_ presented higher affinity to the MGC803 cells with a higher *B*_max_ (5.68 × 10^−19^ mol ligands/cell) and a lower *K*_*d*_ (0.6147 nM), compared to the HT29 cells (*B*_max_ = 1.66 × 10^−19^ mol ligands/cell, Kd = 1.008 nM). These results suggest that MGC803 cells had both higher total EGFR expression level and Cet-F(ab′)_2_ binding affinity than HT29 cells.

### 3.4. In Vitro Biodistribution, SPECT/CT Imaging, and Immunofluorescence

**Figure 6:**
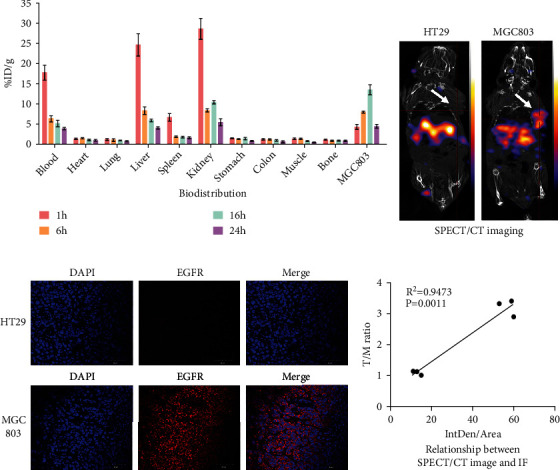
In vitro biodistribution and SPECT/CT imaging. (a) Biodistribution assay demonstrating that ^99m^Tc-MAG_3_-Cet-F(ab′)_2_ uptake in MGC803 tumors peaked at 16 h after injection. (b) ^99m^Tc-MAG_3_-Cet-F(ab′)_2_ SPECT/CT imaging showing significant different radionuclide uptake between an MGC803 tumor and a HT29 tumor (arrow). (c) Immunofluorescence showing the higher EGFR expression in MGC803 tumor slices compared with HT29 tumor slices. (d) Relationship between SPECT/CT image and immunofluorescence.

To quantify ^99m^Tc-MAG_3_-Cet-F(ab′)_2_ uptake at 1, 6, 16, and 24 h after injection, the MGC803 tumor-bearing mouse models underwent in vitro biodistribution studies. ^99m^Tc-MAG_3_-Cet-F(ab′)_2_ uptake in the MGC803 tumors at 1, 6, 16, and 24 h postinjection was 4.28 ± 1.04, 8.00 ± 0.39, 13.51 ± 2.17, and 4.46 ± 0.80%ID/g, respectively, and the T/M ratios were 3.40 ± 1.59, 5.97 ± 0.75, 17.29 ± 5.72, and 11.33 ± 2.62, respectively (Figure 6(a)). At 16 h, the *T*/*L* (tumor to liver, 2.30 ± 0.44), *T*/*K* (tumor to kidney, 1.30 ± 0.15), and *T*/*B* (tumor to blood, 2.78 ± 0.96) ratios were significantly higher than the corresponding results at 1 h (0.18 ± 0.07, 0.15 ± 0.05, 0.25 ± 0.10, respectively), 6 h (0.98 ± 0.15, 0.95 ± 0.05, 1.29 ± 0.26, respectively), and 24 h (1.09 ± 0.09, 0.83 ± 0.13, 1.15 ± 0.09, respectively), respectively. These results indicate that ^99m^Tc-MAG_3_-Cet-F(ab′)_2_ was mainly metabolized by the liver and kidney. Background radioactivity in the stomach, colon, and bone was minimal, which was in agreement with the imaging data. The SPECT/CT imaging (Figure 6(b)) of the subcutaneous tumors demonstrated significantly increased radioactivity accumulation in the MGC803 tumor (high EGFR expression) than in the HT29 tumor (low EGFR expression) (*T*/*M*: 3.21 ± 0.27 vs. 1.09 ± 0.07, *P* < 0.05). Immunofluorescence showed that the MGC803 tumor slices had stronger fluorescence intensity than the HT29 tumor slices, which was consistent with the western blotting and immunocytochemistry results (Figure 6(c)). Spearman correlation analysis clarified the relationship between the T/M ratio on SPECT/CT imaging and the integrated density/area (IntDen/Area) on immunofluorescence (*R*^2^ = 0.9473, *P* < 0.01, Figure 6(d)). These results indicate that ^99m^Tc-MAG_3_-Cet-F(ab′)_2_ is a good tracer for targeting the EGFR expression in tumors.

## 4. Discussion

EGFR is widely expressed in nearly every cancer type, and its high expression in tumors correlates with poor patient outcome [16]. Several studies have demonstrated that patients with digestive tumors benefited from EGFR-targeted therapy using cetuximab [17–19]. For patients with unresectable tumors, it is difficult to evaluate the EGFR expression through pathology. Therefore, noninvasive evaluation of the EGFR expression is particularly important. Cetuximab, an EGFR inhibitor widely used in clinical practice, is suitable for noninvasive evaluation of the EGFR expression [20, 21].

Immuno-SPECT combines the high specificity of antibodies with the high sensitivity of SPECT imaging [22, 23]. Compared with PET, one advantage of SPECT is the price. Typically, achieving the best imaging contrast for a radionuclide-labeled intact antibody (about 150 kDa) requires ≥48 h [8, 10, 24]. Therefore, this renders it unsuitable for imaging radionuclides with short half-lives. For example, for ^99m^Tc (*T*_1/2_ ≈ 6 h), which is most widely used in SPECT imaging, it would be impossible to achieve the best imaging time before dramatic decay when labelling an intact antibody. If a nuclide with a long half-life was used for labelling an intact antibody, which would allow achievement of the best imaging time (>48 h) before dramatic decay, issues regarding high radiation caused by delayed peak tumor uptake and slow clearance would arise, which would hinder the clinical translation. Regarding the radiation issue, the pretargeting imaging strategy is advantageous, allowing the injection of modified mAbs first with a predictable duration for its accumulation to the target site. Then, a small molecule radioligand that can conjugate to the pretargeted mAb is injected for imaging while the redundant radioligand is cleared quickly [25, 26]. The most promising pretargeting methodology is based on inverse electron demand (4 + 2) Diels-Alder (IEDDA) cycloaddition between 1,2,4,5-terazine (Tz) and transcyclooctene (TCO), which has been widely used in tumor imaging studies [26, 27]. Recently, the Tz/TCO-based and cetuximab pretargeted imaging strategy was successfully used for assessing the EGFR expression in colorectal cancer [28]. However, the expensive cost of synthesizing the Tz and TCO molecules and the inconvenience caused by two injections hinder its clinical use. Therefore, the synthesis of a new probe with high specificity and relatively small molecular weight is necessary.

van Dijk et al. [29, 30] prepared Cet-F(ab′)_2_ fragment through pepsin digestion and successfully used it for the imaging EGFR expression of head and neck cancer. Here, we used the IdeS digestion to obtain the Cet-F(ab′)_2_, which is entirely different from pepsin digestion. Compared with pepsin, IdeS is a unique cysteine protease that digests antibodies at a single amino acid site below the hinge region, which is suitable for antibodies from multiple sources (e.g., human, mouse, rabbit, monkey, sheep, chimeric IgG, and Fc fusion protein), with high specificity and rapid reaction time (within 30–60 minutes). van Dijk et al. [29] used pepsin to obtain Cet-F(ab′)_2_ required with a longer reaction time (4 h) and stricter reaction conditions (pH = 3.8). In addition, pepsin would digest many more restriction sites compared to IdeS [31, 32].

The molecular weight of Cet-F(ab′)_2_ is about 100 kDa, which is smaller and therefore leads to quicker clearance, compared to its intact counterpart. Yamaguchi et al. [24] and Perk et al. [33] found that the best imaging time point for intact cetuximab-targeted imaging was 48–72 h, which they confirmed with biodistribution assay and PET imaging. In contrast, the best time point of ^99m^Tc-MAG_3_-Cet-F(ab′)_2_-based imaging as identified through biodistribution assay in the present study was 16 h. Although the molecular weight was reduced only by approximately one-third (150 kDa to 100 kDa), the clearance rate was significantly improved. Therefore, ^99m^Tc is suitable for labeling this tracer, which would certainly reduce patient radiation exposure if translated to the clinic in the future. In addition, immunocytochemistry and SDS-PAGE showed that the Cet-F(ab′)_2_ had similar ability to intact cetuximab to bind to EGFR on tumor cells. Furthermore, the Cet-F(ab′)_2_ had excellent stability in both NS and 1% BSA. In vitro, the Western blotting and the immunocytochemistry assays revealed higher expression of EGFR on the MGC803 cells than the HT29 cells, while the fabricated ^99m^Tc-MAG_3_-Cet-F(ab′)_2_ presented higher affinity to the MGC803 cells with a higher *B*_max_ (5.68 × 10^−19^ mol ligands/cell) and a lower *K*_*d*_ (0.6147 nM), compared to the HT29 cells (*B*_max_ = 1.66 × 10^−19^ mol ligands/cell, Kd = 1.008 nM). These results indicated a good affinity and targeting ability of ^99m^Tc-MAG_3_-Cet-F(ab′)_2_ to EGFR. The biodistribution studies showed that the *T*/*M* ratio peaked at approximately 17.29 ± 5.72 at 16 h after ^99m^Tc-MAG_3_-Cet-F(ab′)_2_ injection. Therefore, we consider 16 h the best imaging time point. In SPECT/CT imaging, the MGC803 tumor had significantly higher ^99m^Tc-MAG_3_-Cet-F(ab′)_2_ uptake than HT29 tumor, which was consistent with in vitro results.

## 5. Conclusion

SPECT/CT imaging using ^99m^Tc-MAG_3_-Cet-F(ab′)_2_ showed rapid and sustained high radionuclide-uptake in EGFR-positive digestive tumors with high image contrast, which indicates the potential for noninvasive evaluation of EGFR expression in tumors.

## Data Availability

The data used to support the findings of this study are available from the corresponding authors upon reasonable request.

## Supplementary Materials

Supplementary Materials Supporting data illustrating the formation and characterization of ^99m^Tc-MAG_3_-Cet-F(ab′)_2_. Figure S1: image of full SDS-PAGE illustrating the quality control of MAG_3_-Cet and MAG_3_-Cet-F(ab′)_2_. Figure S2: the LC-MS results of MAG_3_-Cet and MAG_3_-Cet-F(ab′)_2_. Figure S3: characterization of ^99m^Tc-MAG3-Cet-F(ab′)_2_.

## References

[1] Smyth E. C., Nilsson M., Grabsch H. I., van Grieken N. C., Lordick F. (2020). Gastric cancer. *Lancet*.

[2] Song M., Chan A. T., Sun J. (2020). Influence of the gut microbiome, diet, and environment on risk of colorectal cancer. *Gastroenterology*.

[3] Chong C. R., Janne P. A. (2013). The quest to overcome resistance to EGFR-targeted therapies in cancer. *Nature Medicine*.

[4] Rajaram P., Chandra P., Ticku S., Pallavi B. K., Rudresh K. B., Mansabdar P. (2017). Epidermal growth factor receptor: role in human cancer. *Indian Journal of Dental Research*.

[5] Fornasier G., Francescon S., Baldo P. (2018). An update of efficacy and safety of cetuximab in metastatic colorectal cancer: a narrative review. *Advances in Therapy*.

[6] Cremolini C., Rossini D., Dell’Aquila E. (2019). Rechallenge for patients with RAS and BRAF wild-type metastatic colorectal cancer with acquired resistance to first-line cetuximab and irinotecan: a phase 2 single-arm clinical trial. *JAMA Oncology*.

[7] Forster T., Huettner F. J., Springfeld C. (2020). Cetuximab in pancreatic cancer therapy: a systematic review and meta-analysis. *Oncology*.

[8] Shi D., Zhao S., Jiang W., Zhang C., Liang T., Hou G. (2019). TLR5: A prognostic and monitoring indicator for triple-negative breast cancer. *Cell Death & Disease*.

[9] Kang L., Li C., Rosenkrans Z. T. (2021). Noninvasive evaluation of CD20 expression using64Cu-labeled F(ab’)2fragments of Obinutuzumab in lymphoma. *Journal of Nuclear Medicine*.

[10] Shi D., Liu W., Zhao S., Zhang C., Liang T., Hou G. (2019). TLR5 is a new reporter for triple-negative breast cancer indicated by radioimmunoimaging and fluorescent staining. *Journal of Cellular and Molecular Medicine*.

[11] Menke-Van Der Houven C. W., McGeoch A., Bergstrom M. (2019). Immuno-PET imaging to assess target engagement: experience from89Zr-anti-HER3 mAb (GSK2849330) in patients with solid tumors. *Journal of Nuclear Medicine*.

[12] Hong H., Zhang Y., Orbay H. (2013). Positron emission tomography imaging of tumor angiogenesis with a (61/64)Cu-labeled F(ab')(2) antibody fragment. *Molecular Pharmaceutics*.

[13] Vosjan M. J., Perk L. R., Visser G. W. (2010). Conjugation and radiolabeling of monoclonal antibodies with zirconium-89 for PET imaging using the bifunctional chelate p -isothiocyanatobenzyl- desferrioxamine. *Nature Protocols*.

[14] Kang L., Li C., Yang Q. (2022). 64Cu-labeled daratumumab F (ab’) 2 fragment enables early visualization of CD38-positive lymphoma. *European Journal of Nuclear Medicine and Molecular Imaging*.

[15] Wang Y., Liu X., Hnatowich D. J. (2007). An improved synthesis of NHS-MAG3 for conjugation and radiolabeling of biomolecules with ^99m^Tc at room temperature. *Nature Protocols*.

[16] Landmesser M. E., Raup-Konsavage W. M., Lehman H. L., Stairs D. B. (2020). Loss of p120ctn causes EGFR-targeted therapy resistance and failure. *PLoS One*.

[17] Yang G., Huang L., Jia H. (2021). NDRG1 enhances the sensitivity of cetuximab by modulating EGFR trafficking in colorectal cancer. *Oncogene*.

[18] Shi M., Shi H., Ji J. (2014). Cetuximab inhibits gastric cancer growth in vivo, independent of KRAS status. *Current Cancer Drug Targets*.

[19] Yoong J., Michael M., Leong T. (2011). Targeted therapies for gastric cancer. *Drugs*.

[20] McKnight B. N., Kim S., Boerner J. L., Viola N. T. (2020). Cetuximab PET delineated changes in cellular distribution of EGFR upon dasatinib treatment in triple negative breast cancer. *Breast Cancer Research*.

[21] van Helden E., Elias S., Gerritse S. (2020). Zr-cetuximab PET/CT as biomarker for cetuximab monotherapy in patients with RAS wild-type advanced colorectal cancer. *European Journal of Nuclear Medicine and Molecular Imaging*.

[22] Heskamp S., van Laarhoven H. W. M., Molkenboer-Kuenen J. D. M. (2010). ImmunoSPECT and immunoPET of IGF-1R expression with the radiolabeled antibody R1507 in a triple-negative breast cancer model. *Journal of Nuclear Medicine*.

[23] Guo X., Zhu H., Liu T. (2019). Development of 99mTc-conjugated JS001 antibody for in vivo mapping of PD-1 distribution in murine. *Bioorganic & Medicinal Chemistry Letters*.

[24] Yamaguchi A., Achmad A., Hanaoka H. (2019). Immuno-PET imaging for non-invasive assessment of cetuximab accumulation in non-small cell lung cancer. *Cancer*.

[25] Boerman O. C., van Schaijk F. G., Oyen W. J., Corstens F. H. (2003). Pretargeted radioimmunotherapy of cancer: progress step by step. *Journal of Nuclear Medicine*.

[26] Rossin R., Lappchen T., van den Bosch S. M., Laforest R., Robillard M. S. (2013). Diels-Alder reaction for tumor pretargeting: in vivo chemistry can boost tumor radiation dose compared with directly labeled antibody. *Journal of Nuclear Medicine*.

[27] García M. F., Zhang X., Shah M. (2016). 99mTc-bioorthogonal click chemistry reagent for in vivo pretargeted imaging. *Bioorganic & Medicinal Chemistry*.

[28] Qiu L., Lin Q., Si Z. (2021). A pretargeted imaging strategy for EGFR-positive colorectal carcinoma via modulation of Tz-radioligand pharmacokinetics. *Molecular Imaging and Biology*.

[29] van Dijk L. K., Hoeben B. A., Kaanders J. H., Franssen G. M., Boerman O. C., Bussink J. (2013). Imaging of epidermal growth factor receptor expression in head and neck cancer with SPECT/CT and ^111^In-labeled cetuximab-F(ab')2. *Journal of Nuclear Medicine*.

[30] van Dijk L. K., Yim C. B., Franssen G. M. (2016). PET of EGFR with 64Cu-cetuximab-F(ab’)2 in mice with head and neck squamous cell carcinoma xenografts. *Contrast Media & Molecular Imaging*.

[31] Nowak C., Patel R., Liu H. (2018). Characterization of recombinant monoclonal IgG2 antibodies using LC-MS and limited Lys-C digestion. *Journal of Chromatography. B, Analytical Technologies in the Biomedical and Life Sciences*.

[32] Falkenburg W. J., Van Schaardenburg D., Ooijevaar-de Heer P. (2017). Anti-hinge antibodies recognize IgG subclass- and protease-restricted Neoepitopes. *Journal of Immunology*.

[33] Perk L. R., Visser G. W. M., Vosjan M. J. W. D. (2005). 89Zr as a PET surrogate radioisotope for scouting biodistribution of the therapeutic radiometals (90)Y and (177)Lu in tumor-bearing nude mice after coupling to the internalizing antibody cetuximab. *Journal of Nuclear Medicine*.

