# Supplementary material for: Noninvasive Evaluation of EGFR Expression of Digestive Tumors Using 99mTc-MAG3-Cet-F(ab′)2-Based SPECT/CT Imaging
Source: Mol Imaging. 2022 Jun 24;2022:3748315. doi: 10.1155/2022/3748315 (PMC9281432; doi:10.1155/2022/3748315)
Supplement: Supplementary Materials — Supporting data illustrating the formation and characterization of 99mTc-MAG3-Cet-F(ab′)2. Figure S1: image of full SDS-PAGE illustrating the quality control of MAG3-Cet and MAG3-Cet-F(ab′)2. Figure S2: the LC-MS results of MAG3-Cet and MAG3-Cet-F(ab′)2. Figure S3: characterization of 99mTc-MAG3-Cet-F(ab′)2. [file 3748315.f1.docx]

**Title:** Supporting data illustrating the formation and characterization of ^99m^Tc-MAG_3_-Cet-F(ab')_2_

**
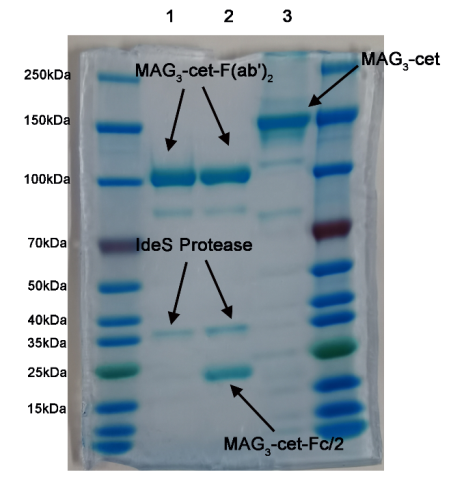
**

**Figure S1: Image of full SDS-PAGE illustrating the quality control of MAG_3_-Cet and MAG_3_-Cet-F(ab')_2_.** The lane 1 represents the MAG_3_-Cet-F(ab')_2_ after digestion of MAG_3_-Cet with IdeS protease and reaction with protein A beads. The lane 2 represents the mixture of MAG_3_-Cet-F(ab')_2_, MAG_3_-Cet-Fc/2 and IdeS protease after digestion of MAG_3_-Cet with IdeS protease but without reaction with protein A beads. The lane 3 represents the MAG_3_-cet. The amount of IdeS protease is less than 3% of the total amount, and does not chelate with NHS-MAG_3_, so it will not affect the radiochemical purity of the product.


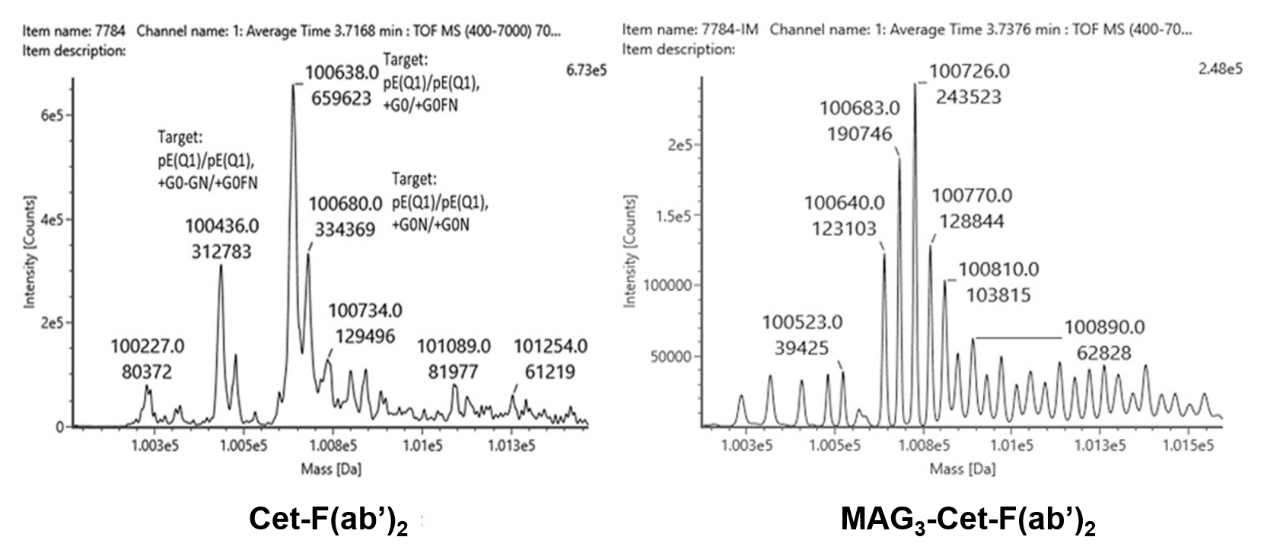


**Figure S2: The LC-MS results of MAG_3_-Cet and MAG_3_-Cet-F(ab')_2_.** The sample protein (~20ug) was loaded to LC-MS and conjugation ratio was calculated based on the peak abundance of the deconvoluted mass. LC-MS result and the calculation of the number of MAG_3_ attached per antibody were shown below.


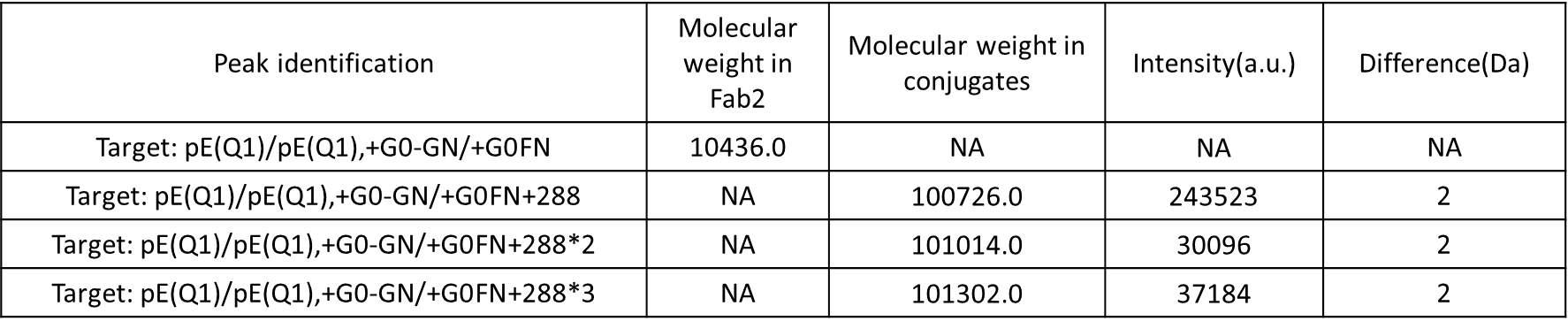


The peak (Target: pE(Q1)/pE(Q1),+G0-GN/+G0FN) in F(ab')_2_ were used to calculate the conjugation ratio in F(ab')_2_-MAG_3_.

Overall intensity of Target: pE(Q1)/pE(Q1),+G0-GN/+G0FN related peak is 243523+ 30096+37184=310803

Conjugation of MAG_3_ is 3*37184/310803++2*30096/310803+1*243523/310803=1.336


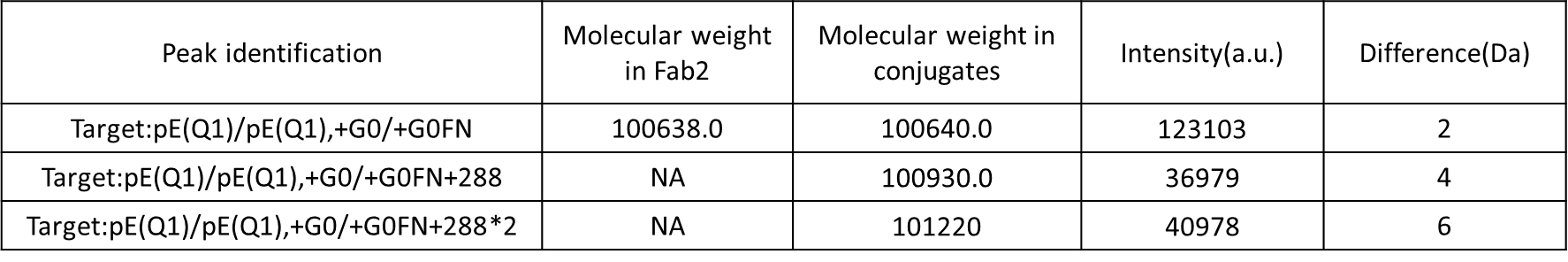


The peak (Target: pE(Q1)/pE(Q1),+G0/+G0FN)in F(ab')_2_ were used to calculate the conjugation ratio in F(ab')_2_-MAG_3_.

Overall intensity of Target: pE(Q1)/pE(Q1),+G0/+G0FN related peak is 123103+ 36979+ 40978=201620

Conjugation of MAG_3_ is 2*40978/201620+1*36979/201620=0.5914


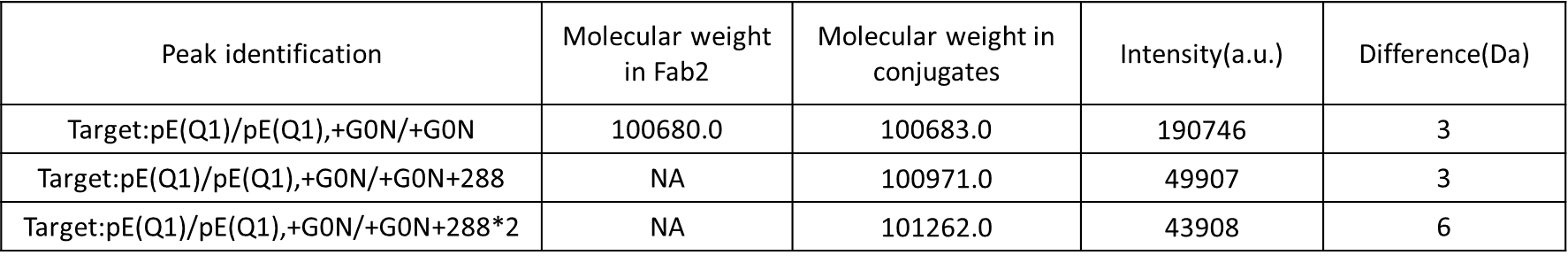


The peak (Target: pE(Q1)/pE(Q1),+G0N/+G0N)in F(ab')_2_ were used to calculate the conjugation ratio in F(ab')_2_-MAG_3_.

Overall intensity of Target: pE(Q1)/pE(Q1),+G0N/+G0N related peak is 190746+ 49907+ 43908=284561

Conjugation of MAG_3_ is 2*43908/284561+1*49907/284561=0.4840 The main peak intensity in F(ab')_2_ is 312783+659623+334369=1306775 (Target: pE(Q1)/pE(Q1),+G0-GN/+G0FN+Target: pE(Q1)/pE(Q1),+G0/+G0FN+Target: pE(Q1)/pE(Q1),+G0N/+G0N)

Thus the overall conjugation ratio is 312783/1306775*1.336+659623/1306775*0.5914

+334369/1306775*0.4840=0.74


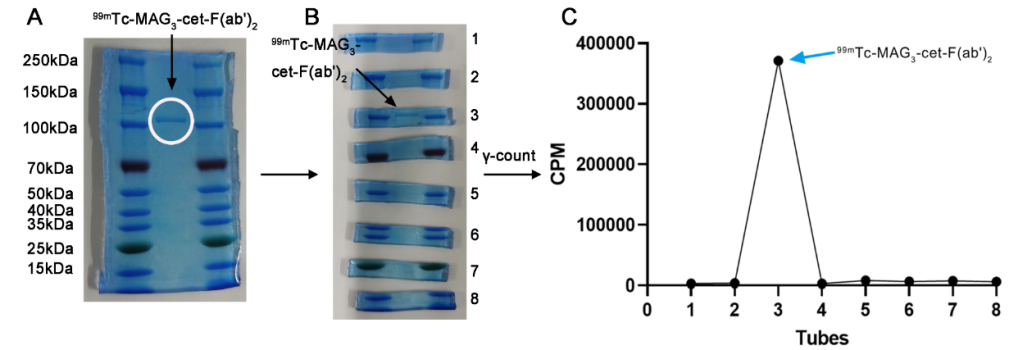


**Figure S3: Characterization of ^99m^Tc-MAG_3_-Cet-F(ab')_2_. A**, western blot image of the ^99m^Tc-MAG_3_-Cet-F(ab')_2_ based on Coomassie Brilliant Blue staining showing the position of ^99m^Tc-MAG_3_-Cet-F(ab')_2_ (white circle). **B,** the entire SDS-PAGE from **A** was divided to 8 segments, of which the radioactivity counts were tested with a gamma count, as shown in **C**, indicating a high radiolabeling rate of ^99m^Tc-MAG_3_-Cet-F(ab')_2_.
